# Removal of toxic lead from aqueous solution using a low-cost adsorbent

**DOI:** 10.1038/s41598-023-29674-x

**Published:** 2023-02-25

**Authors:** Mohammad Hadi Dehghani, Sahar Afsari Sardari, Mojtaba Afsharnia, Mehdi Qasemi, Mahmoud Shams

**Affiliations:** 1 Department of Environmental Health Engineering, School of Public Health, Tehran University of Medical Sciences, Tehran, Iran; 2 Institute for Environmental Research, Center for Solid Waste Research, Tehran University of Medical Sciences, Tehran, Iran; 3 Department of Chemical Engineering, Faculty of Engineering, Razi University, Kermanshah, Iran; 4 Department of Environment Health Engineering, School of Health, Infectious Diseases Research Center, Gonabad University of Medical Sciences, Gonabad, Iran; 5 PhD Student of Environment Health Engineering, Student Workgroup of Social Development and Health Promotion Research Center, Gonabad University of Medical Sciences, Gonabad, Iran; 6 Social Determinants of Health Research Center, Mashhad University of Medical Sciences, Mashhad, Iran

**Keywords:** Environmental sciences, Chemistry

## Abstract

Valorization of waste materials and byproducts as adsorbents is a sustainable approach for water treatment systems. Pottery Granules (PG) without any chemical and thermal modification were used as a low-cost, abundant, and environmentally benign adsorbent against Pb(II), the toxic metal in drinking water. The porous structure and complex mineral composition of PG made it an efficient adsorbent material for Pb(II). The effect of key physicochemical factors was investigated to determine the significance of contact time, PG dose, pH, solution temperature, and coexisting ions, on the process. Pb(II) removal increased by PG dose in the range of 5–15 g/L, and agitation time from 5 to 60 min. Increasing Pb(II) concentration led to a drop in Pb(II) removal, however, adsorption capacity increased significantly as concentration elevated. Pb(II) removal also increased significantly from ~ 45% to ~ 97% by pH from 2 to 12. A ~ 20% improvement in Pb(II) adsorption after rising the solution temperature by 30˚C, indicated the endothermic nature of the process. The sorption was described to be a favorable process in which Pb(II) was adsorbed in a multilayer onto the heterogeneous PG surface. The qmax of 9.47 mg/g obtained by the Langmuir model was superior among many reported low-cost adsorbents. The Pb(II) adsorption was described well by the Pseudo- first-order kinetic model. Na^+^, Mg^2+^, Ca^2+^, Cd^2+^, and Zn^2+^ showed a negligible effect on Pb(II) adsorption. However, the presence of Mn^2+^ and Fe^2+^ significantly hindered the process efficacy. In conclusion, the use of waste material such as PG against Pb(II) is a viable option from the economic and effectiveness points of view.

## Introduction

Environmental pollution with heavy metals could impose significant impacts on human health and the environment due to the non-biodegradable and accumulative nature^1–3^. Contamination of water resources by emerging contaminants such as heavy metals by industrial discharges is a serious challenge, especially in developing countries^4–7^. Mining, smelting, fossil fuels combustion, solid waste incineration, manufacturing batteries, paints, electronic, ceramics, and glass industries are among the anthropogenic sources of heavy metals. Natural phenomena such as forest fires, volcanos, mineral weathering, and erosion, also are responsible for the occurrence of heavy metals^8–10^.

Pb(II) is a non-essential, toxic, and enzyme inhibitor heavy metal with an MCLG of zero^11^. Studies endorsed the intake of Pb(II) as an etiological factor for serious damage to the central nervous system, reproductive system, liver, and kidney^12–15^. However Pb(II) could penetrate the body by different routes, the ingestion through drinking water was known as the main pathway for Pb(II) intake^16^. Even in developed countries, there is a growing concern for lead exposure by the presence of Pb(II) at levels exceeding the drinking water standard^17,18^. Two general strategies exist to abate the negative consequences of this toxic contaminant in drinking water, corrosion control, and removal. USEPA suggested corrosion control as a “treatment technology” to prevent leaching noxious Pb(II) from the old water plumbing^19^. Another option, Pb(II) removal from contaminated streams, was studied extensively in recent years.

Of the physical, chemical, and biological technologies that exist to abate heavy metals and other contaminants, adsorption is known as a promising option due to the simplicity of design, ease of operation, and efficient removal of the contaminants in trace levels^20,21^. The process is especially viable when it is accomplished with available and low-cost adsorbents. Seeking cheap, natural, abundant, and environmentally friendly adsorbents for heavy metals was being an interest in different studies^22–26^. Low-cost zeolite^27^, Eupatorium Adenophorum spreng^28^, dry desulfurization slag^29^, manganoxide^30^, reed root, sawdust, seaweed^31^, fly ash^32,33^, and nopal cladodes^34^, were successfully employed against Pb(II) in a lab or full-scale treatment units. Moreover, Lingamdinne et al. used Lonicera japonica flower powder and the magnetized form as low-cost, eco-friendly, and efficient adsorbents against Co(II), Pb(II), and Co(II)^35,36^.

Valorization of waste materials and byproducts into a useful adsorbent is a promising option to prevent waste generation and to have an economic adsorption system^37^. In this context, the use of pottery wastes produced in a huge amount during the production and transportation of ceramic industries would have environmental and economic advantages.

Pottery waste produced in different countries has been studied for environmental remediation and as a structural material^38^. Bouatay et al. valorized Tunisian Pottery Clay as a low-cost material for Basic dyes and realized the adsorption capacity was in the range of commercial powdered activated carbon^39^. Hao et al. prepared a carbon/pottery composite for fluoride uptake and demonstrated a good adsorption efficacy of 88.56% and an adsorption capacity of 2.214 mg/g^40^. Khazali et al. highlighted the presence of large deposits of clay minerals in different locations in Jordan and studied the possibility of Jordanian pottery materials for copper(II) adsorption^41^.

Herein, the pottery granules (PG) which are environmentally benign, low cost and abundant waste material, were studied against Pb(II) in the aqueous medium. After minimal preparation steps, PG was characterized and used in a batch sorption system to evaluate the effect of key operating variables i.e. pH, adsorbent dose, and the presence of competitive ions, on Pb(II) adsorption. The non-linear kinetic and equilibrium models fitted the experiments to elucidate the mechanisms that govern the adsorption. Moreover, the economic viability of the adsorption to scale up to the real treatment system is justified.

## Material and methods

### Adsorbent preparation and characterization

Except for pottery granules (PG), all the chemicals used in the study were of analytical grade and obtained from Merck. Pb(II) solutions with desired concentration prepared from a 100 mg/L stock solution prepared by Pb(NO_3_)_2_. Pottery wastes were obtained from the local pottery manufactories, washed thoroughly with deionized water, and oven-dried at 70 °C for 24 h. Pottery wastes were then crushed in a ball mill, and sieved with a stainless steel #20 (0.84 mm) screener to obtain a uniform granular particle size. The structural characteristics of PG were analyzed by x-ray diffraction (XRD) and scanning electron microscope (FESEM) analysis.

The pH at zero point of charge (pHzpc) of PG was determined to determine the mechanism of Pb(II) adsorption. 0.1 g of PG was added to the solutions with initial pH in the range of 2–10 (pHi). After mixing for 24 h, the final pH was measured as pHf. The pHzpc was then estimated from the intercept of ΔpH (pHi—pHf) vs pHi. The pHzpc of PG was determined to be ~ 8.7.

### Adsorption experiments

Adsorption experiments were carried out in an incubator shaker at 250 rpm at 23 ± 1 °C. Similar to temperature, pH was not controlled during the experiments and was ~ 5.6. Samples were taken at predetermined time intervals for Pb(II) measurement using a furnace atomic absorption spectroscopy (Varian AA240FS). The responses for the process, Pb(II) removal efficiency (Re, %), and PG adsorption capacity (q, mg/g) were calculated from Eqs. (1) and (2), respectively.

1 $${\varvec{R}}{\varvec{e}} = \frac{({{\varvec{C}}}_{0}-{{\varvec{C}}}_{{\varvec{t}}})}{{{\varvec{C}}}_{0}}\times 100$$

2 $${\varvec{q}} = \frac{({{\varvec{C}}}_{0}-{{\varvec{C}}}_{{\varvec{t}}})({\varvec{V}})}{{\varvec{m}}}\times 100$$

where C0 and Ct (mg/L) are the concentration of Pb(II) at time = 0, and any time, V (L) is the solution volume, and m (g) is the mass of PG. To obtain the PG capacity at the equilibrium, Eq. (2) was used where q and Ct were named qe and Ce, respectively.

The coefficient of determination (R^2^), Adjusted R-square (R^2^_adj_), and the sum of squares error (SSE) are useful statistical parameters to determine the suitability and accuracy of the models. As R^2^ value is close to unity, the model describes the data more accurately. This is also the case for R^2^_adj_, however, R^2^_adj_ value does not always get close to unity by the number of inputted variables. Another parameter, the sum of square error (SSE), reveals the difference between data for the experiments and their corresponding values estimated by the model. The model with a lower SSE would have a more precise estimation.

Nonlinear regression models were applied to the experimental data for kinetic and equilibrium studies. Compared to linear forms, nonlinear regression provides a more precise estimation of the model terms. Nonlinear models for isotherm and kinetic studies and the statistical parameters used for the suitability of the models are summarized in Table 1.

**Table 1:** The models and statistical parameters used in this study 42,43.

Type of equation	Nonlinear form	Parameters
Isotherm models		
Langmuir	${q}_{e}=\frac{{{Q}_{m}K}_{L}{C}_{e}}{{1+K}_{L}{C}_{e}}$	C_e_ = adsorbate equilibrium concentration (mg/L) q_e_ = adsorption capacity at equilibrium (mg/g) Q_m_ = monolayer coverage capacity (mg/g) K_L_ = Langmuir isotherm constant (L/mg)
Freundlich	$q_{e} = K_{f} C_{e}^{{1/n}}$	K_f_ = Freundlich isotherm constant(mg^1−(1/n)^ L^1/n^ g^−1^) n = adsorption intensity
Sips	${\mathrm{q}}_{\mathrm{e}}=\frac{{\mathrm{q}}_{{\mathrm{m}}_{\mathrm{s}}}{\mathrm{K}}_{\mathrm{s}}{\mathrm{C}}_{\mathrm{e}}^{{\mathrm{m}}_{\mathrm{s}}}}{1+{\mathrm{K}}_{\mathrm{s}}{\mathrm{C}}_{\mathrm{e}}^{{\mathrm{m}}_{\mathrm{s}}}}$	${\mathrm{q}}_{{\mathrm{m}}_{\mathrm{s}}}$= Sips maximum adsorption capacity (mg/g) K_S_ = Sips equilibrium constant ${(\mathrm{L}/\mathrm{mg})}^{{\mathrm{m}}_{\mathrm{s}}}$ m_S_ = Sips model exponent
Hill	${\mathrm{q}}_{\mathrm{e}}=\frac{{\mathrm{qHC}}_{\mathrm{e}}^{nH}}{{\mathrm{K}}_{\mathrm{D}}+{\mathrm{C}}_{\mathrm{e}}^{nH}}$	qH = Maximum uptake saturation (mg/L) ${\mathrm{C}}_{\mathrm{e}}^{nH}$, ${\mathrm{K}}_{\mathrm{D}}$= Hill constants
Redlich-Peterson	${\mathrm{q}}_{\mathrm{e}}=\frac{{\mathrm{K}}_{\mathrm{RP}}{\mathrm{C}}_{\mathrm{e}}}{1+{\mathrm{a}}_{\mathrm{RP}}{\mathrm{C}}_{\mathrm{e}}^{\mathrm{g}}}$	K_RP_ = Redlich-Peterson isotherm constant(L/g) a_RP_ = Redlich-Peterson model constant(mg/L)^-g^ g = Redlich-Peterson model exponent
Khan	${\mathrm{q}}_{\mathrm{e}}=\frac{{\mathrm{q}}_{\mathrm{s}}{b}_{k}}{{(1+{\mathrm{b}}_{\mathrm{k}}{C}_{e})}^{ak}}$	bK = is the Khan model constant aK = Khan model exponent
Kinetic models		
Pseudo first-order	$\frac{{\mathrm{dq}}_{t}}{{d}_{t}}={k}_{1}({q}_{e}-{q}_{t})$	q_e_ = adsorption capacity at equilibrium (mg/g) q_t_ = adsorption capacity at any time (mg/g) ${k}_{1}$= Rate constant min^−1^
Pseudo second-order	$\frac{{\mathrm{dq}}_{t}}{{d}_{t}}={k}_{2}{({q}_{e}-{q}_{t})}^{2}$	${k}_{2}$= Rate constant (g/(mg min))
Intraparticle diffusion kinetic	${q}_{t}={K}_{t}\times {t}^{0.5}+{C}_{t}$	${K}_{t}$= Rate constant (mg/(g min))
Statistical parameter		
Coefficient of determination (R^2^)	${R}^{2}=1-\frac{\sum_{i=1}^{n}{({y}_{ip}-{y}_{i})}^{2}}{\sum_{i=1}^{n}{({y}_{ip}-\overline{y })}^{2}}$	${y}_{ip}$=The predicted value by the model y_i_ = The observed value $\overline{y }$ =Mean of observed value ${n}_{p}$=The number of variables in the model
Adjusted R-square (R^2^adj)	${R}_{adj}^{2}=1-(\frac{n-1}{1-\left({n}_{p}+1\right)})(1-{R}^{2})$	
Sum of squares error (SSE)	$SSE=\sum_{i=1}^{n}{({y}_{ip}-{y}_{i})}^{2}$	

## Results and discussion

### Adsorbent characteristics

**Figure 1:**
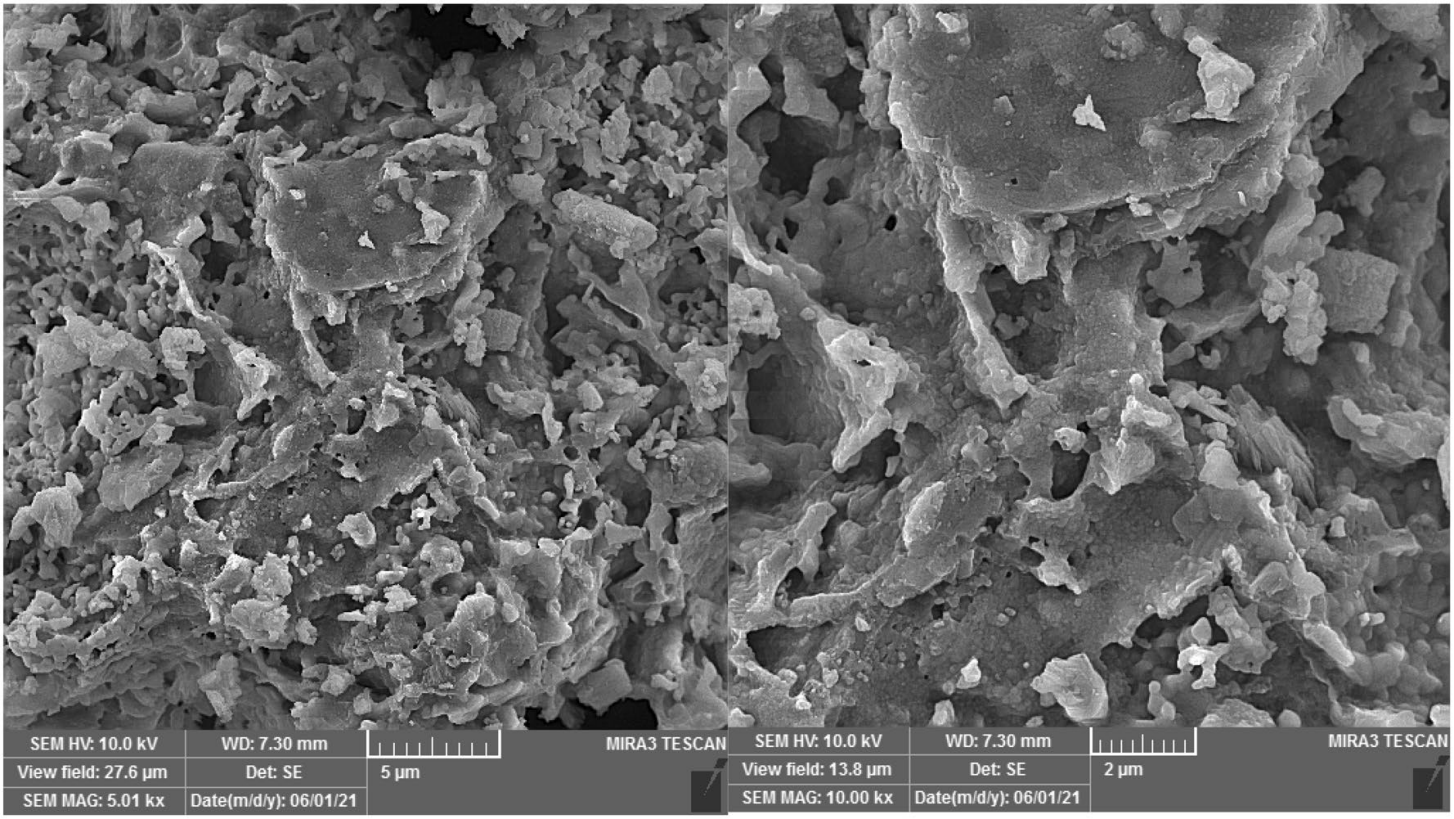
FESEM of PG adsorbent with two magnification.

**Figure 2:**
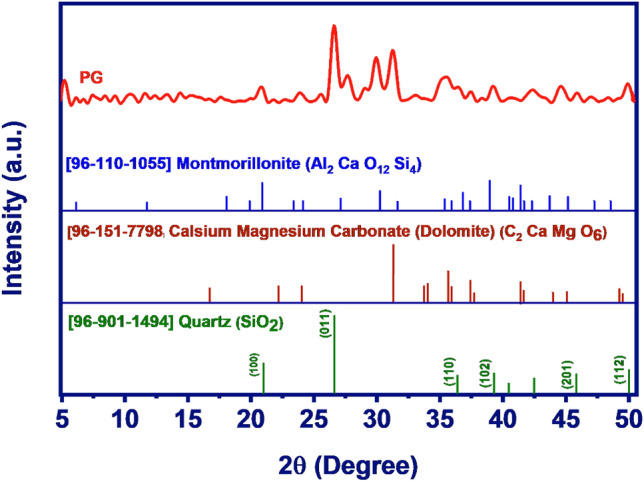
XRD pattern of PG used for Pb(II) removal.

Figure 1 shows the FESEM images of PG with two magnifications. As seen, PG is composed of aggregations of amorph particles. Moreover, the presence of frequent voids and pores in the figures reflected the porous nature of the structure. The structural composition and crystalline phases of PG were also determined by XRD and the results are presented in Fig. 2. As seen, the chemical composition of PG mainly composed of Al_2_ Ca O_12_ Si_4_ (52%), C_2_ Ca Mg O_6_ (31.4%), and SiO_2_ (16.2%).

### Effect of PG dose, mixing time, and Pb(II) concentration

**Figure 3:**
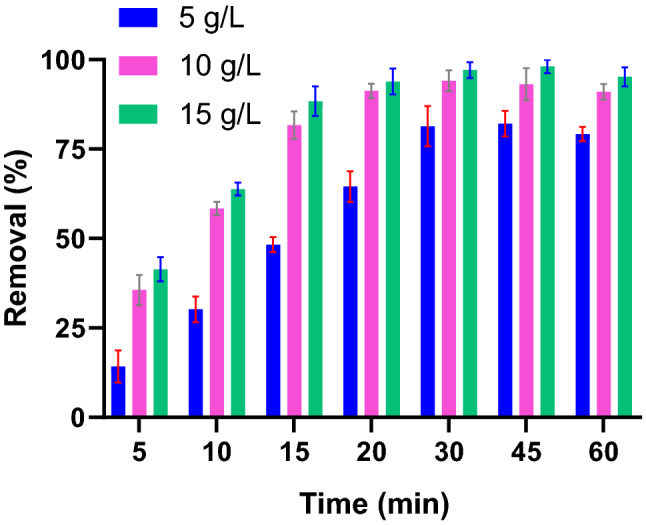
Pb(II) removal as a function of adsorbent dose (Pb(II): 10 mg/L).

Contact time and adsorbent dose are impportant variables that determine the economic viability of the process in real treatment plants. Pb(II) removal was determined as a function of PG masses added to the solutions. As shown in Fig. 3, Pb(II) adsorption increased significantly by dose from 5 to 15 g/L. Similar findings were observed for antibiotics^44^ and Pb(II)^45^ attributed to the frequently available sorption sites for contaminants under elevated doses. The figure also shows that the removal was not changed significantly when the adsorbent dose increased by 10 g/L. Pb(II) uptake is also dependent on the mixing time for all adsorbent doses. For instance, a sharp increase in adsorption of Pb(II) from about 35% to ~ 93% was observed in Fig. 3 by time in the range of 5–60 min. It can also be deducted from the figure that the PG rapidly uptake Pb(II) and the equilibrium was almost reached within 30 min. The fast adsorption is of economic and operational advantages, as the treatment unit could operate in small size and high rate mode.

**Figure 4:**
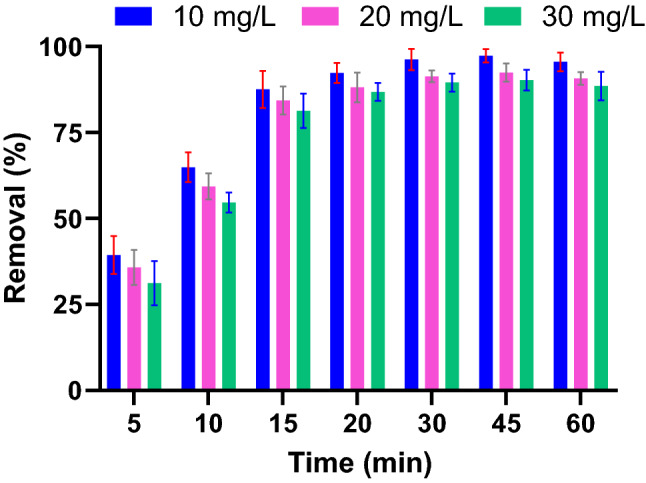
Pb(II) removal as a function of Pb(II) concentrations. (PG: 10 g/L).

**Figure 5:**
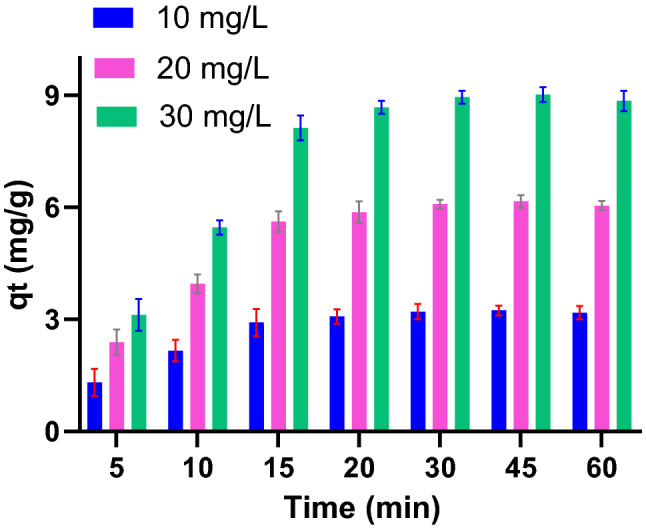
Adsorbent capacity (qt) as a function of Pb(II) concentration (PG: 10 g/L).

Figure 4 plotted the removal efficiency of PG for Pb(II) under the different initial concentrations. The figure clearly showed a declining in Pb(II) adsorption by increasing the metal concentration. This behavior could be explained by the higher competition developed for Pb(II) ions to adsorb on limited sorption sites that exist on the surface. The adsorbent capacity (q_t_) for Pb(II), on the other hand, increased significantly by increasing Pb(II) ions in the solution. As indicated in Fig. 5, increasing adsorbent capacity by Pb(II) concentration reflected an efficient utilization of adsorbent sites.

### Effect of pH

**Figure 6:**
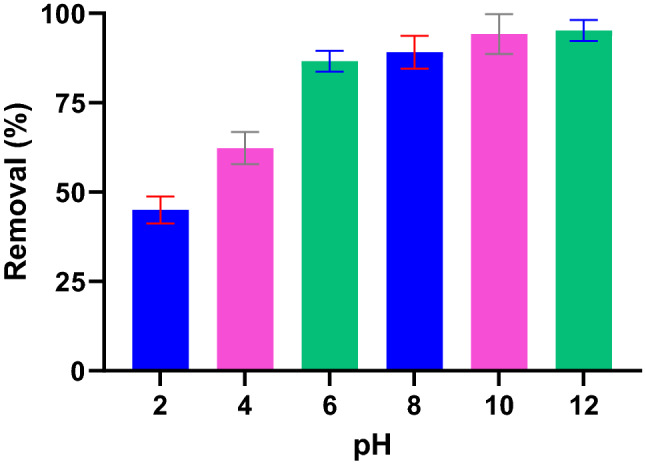
Effect of pH on Pb(II) adsorption (PG: 10 g/L, Pb(II): 10 mg/L).

pH is known as a key physicochemical variable that controls the adsorption process by directing the electrostatic interactions by changing the ionic state of the chemicals in water and also the adsorbent surface charge^43^. The effect of solution pH was studied in the range of 2–12. As presented in Fig. 6, Pb(II) adsorption was favorable at alkaline conditions and Pb(II) removal decreased from ~ 97 to ~ 45% when pH decreased from 12 to 2. Low pH was not favored Pb(II) adsorption, however, the removal rate remained above 60% at pH 4. The mechanism of Pb(II) adsorption as a function of pH could explain by the ionic species of Pb(II) and PG surface charge. The pH_ZPC_ of PG was determined to be ~ 8.7 that means at pH < 8.7 the surface of PG is positively charged. Under the strong acidic condition, the H_3_O^+^ and Pb^2+^ species dominated in the solution, and a net repulsive force developed between these cations and' PG^+^ surface. Studies on clay^46^ and granulated blast-furnace slag^47^ also showed that Pb(II) removal depends strongly on the pH of solutions and removal dropped dramatically as pH decreased.

### Effect of temperature

**Figure 7:**
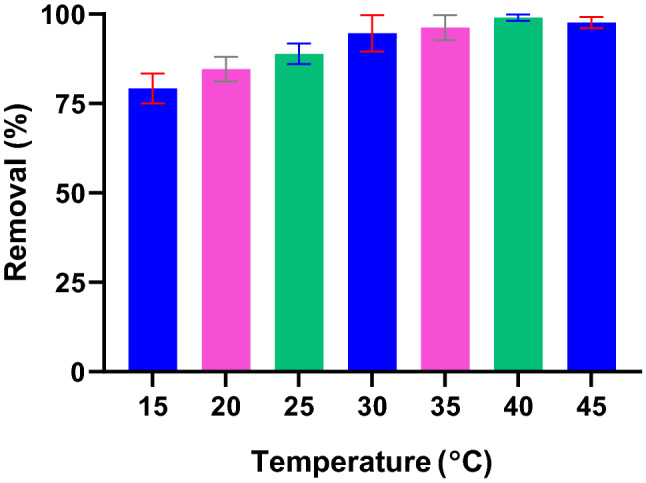
Effect of temperature on Pb(II) adsorption (PG: 10 g/L, Pb(II): 10 mg/L).

Study the solution temperature in sorption systems is essential since it is a common phenomenon in real water treatment systems. To realize how solution temperature affects Pb(II) removal, experiments were carried out at temperatures ranging from 15 to 45 °C. As seen in Fig. 7, Pb(II) adsorption increases by 18% when the temperature raised in the studied range.

An improved adsorption performance of Pb(II) onto PG by increasing the solution temperature is indicative of the endothermic nature of the process. Adsorption of Pb(II) ions as a function of temperature was studied for different adsorbents in the literature. Hefne et al. studied the Pb(II) adsorption onto natural bentonite in the range of 293–313 K and found the process is endothermic and spontaneous^48^. Wan Ngah et al. studied the Pb(II) and Cu(II) removal by natural composite chitosan-tripolyphosphate beads and realized the adsorption of both metals are endothermic^49^. Pb(II) adsorption onto Turkish kaolinite clay as a natural and abundant mineral studied by Sari et al. and found to be exothermic^50^.

### Kinetic study

**Figure 8:**
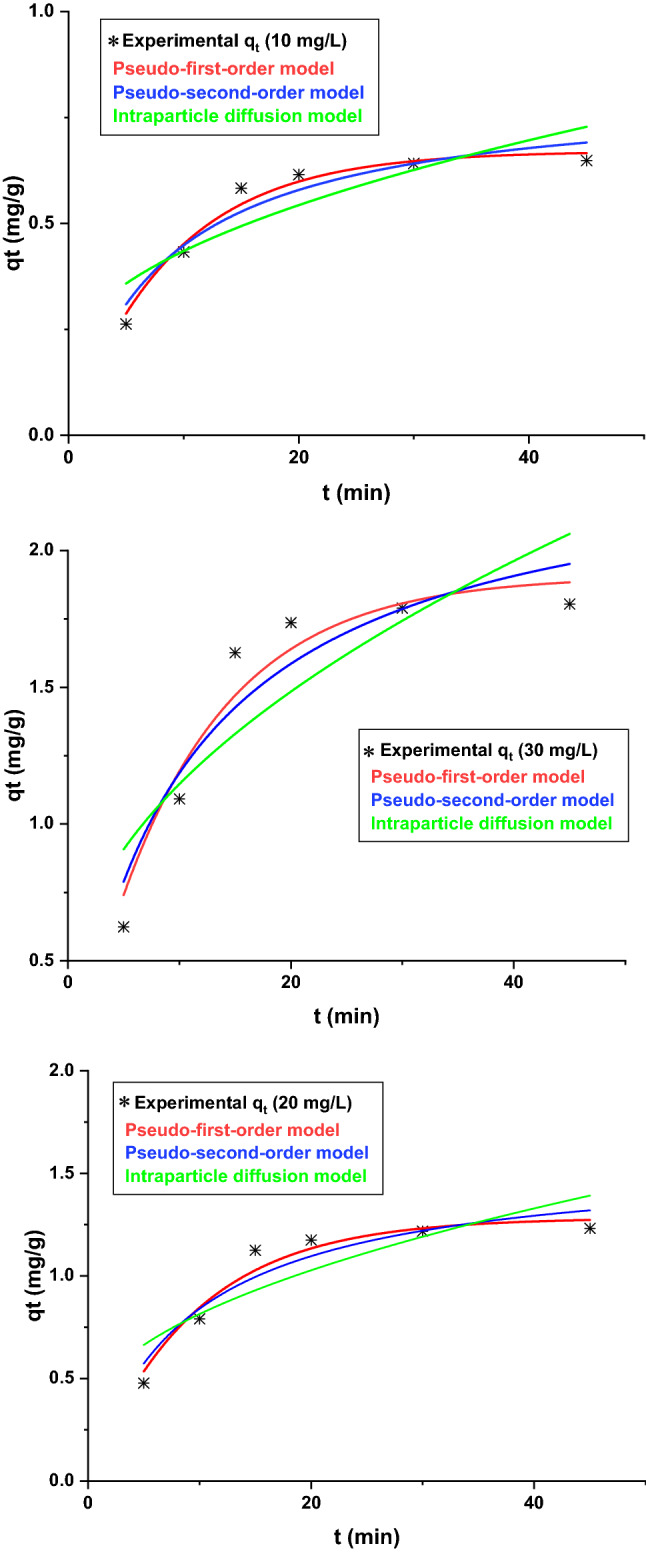
Fitting the three widely kinetic models for Pb(II) adsorption by Pb(II).

Kinetic and Isotherm models could give essential parameters for the design and operation of adsorption systems. The capacity of an adsorbent for a specific contaminant and the rate of sorption were addressed in equilibrium and kinetic modeling, respectively. To model the kinetics of Pb(II) adsorption, the removal efficiency was monitored as a function of mixing time and initial metal concentration (10, 20, and 30 mg/L). The nonlinear forms of pseudo-first-order, pseudo-second-order, and intraparticle diffusion (Weber- Morris) models described in Table 1 were used to figure out the best kinetic model^20,51^. The graphical representation of kinetic modeling is presented in Fig. 8. Kinetic and statistical parameters are summarized in Table 2. As seen in the table, the pseudo-first-order model has a higher R^2^ and ${\mathbf{R}}_{\mathbf{A}\mathbf{d}\mathbf{j}}^{2}$ and smaller RSS that indicate the suitability of this model to describe the kinetic data.

**Table 2:** Kinetic parameters for Pb(II) adsorption by PG.

Concentration (mg/L)	10	20	30
q_e_, exp (mg/g)	0.64	1.23	1.80
Pseudo-first order model			
q_e_ (mg/g)	0.67	1.28	1.9
k_1_ (min^-1^)	0.11	0.1	0.11
${\mathrm{R}}_{\mathrm{Adj}}^{2}$	0.96	0.95	0.93
R^2^	0.97	0.94	0.94
RSS	0.00	0.02	0.06
Pseudo-second order model			
q_e_ (mg/g)	0.81	1.57	2.39
k_2_ (g/mg min)	0.14	0.07	0.04
${\mathrm{R}}_{\mathrm{Adj}}^{2}$	0.90	0.88	0.87
R^2^	0.92	0.90	0.89
RSS	0.00	0.04	0.12
Interparticle diffusion model			
k_3_	0.08	0.16	0.25
C	0.17	0.29	0.33
${\mathrm{R}}_{\mathrm{Adj}}^{2}$	0.69	0.67	0.67
R^2^	0.75	0.73	0.73
RSS	0.03	0.12	0.30

### Equilibrium study

**Figure 9:**
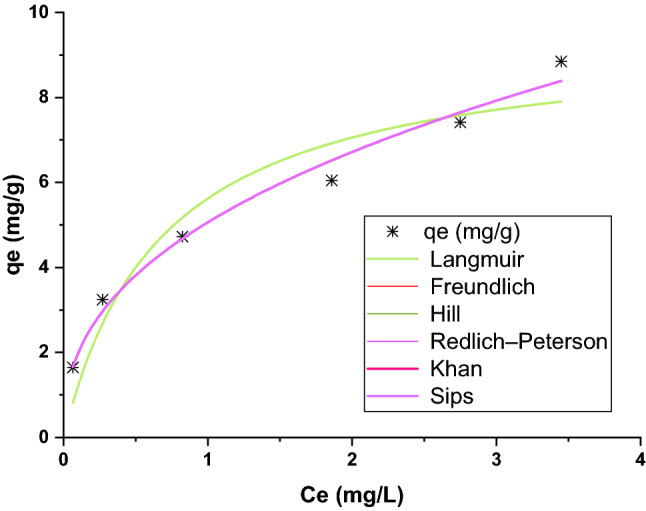
Fitting the six widely isotherm models for Pb(II) adsorption by Pb(II).

As discussed earlier, equilibrium studies are important in the economy of sorption because it determines the mass of adsorbent required to treat contaminated stream^52^. The data from equilibrium experiments were fitted to the most frequently used models. The Langmuir, Freundlich, Sips, Redlich-Peterson, Hill, and Khan isotherm models as listed in Table 1 fitted to equilibrium data and the results presented in Fig. 9. Also, Table 3 summarized the isotherm parameters for Pb(II) adsorption by PG. As seen, R2 and R_adj_^2^ are closest to unity and RSS in minimum for the Freundlich model. The Freundlich equation is an empirical equation that described the adsorption to occur as multilayers onto a heterogeneous surface. This finding endorsed the porous structure of PG as discussed in the characterization section.

**Table 3:** Nonlinear equilibrium model parameters for Pb(II) adsorption.

Isotherm	Parameters	Values
Langmuir	b (L mg^‒1^)	1.46
	Q_max_ (mg/g)	9.47
	${\mathrm{R}}_{\mathrm{Adj}}^{2}$	0.89
	R^2^	0.91
	RSS	2.89
Freundlich	K_f_ (mg g^‒1^) (mg^‒1^)^1/n^	5.06
	n	2.45
	${\mathrm{R}}_{\mathrm{Adj}}^{2}$	0.98
	R^2^	0.98
	RSS	0.56
Sips	q_ms_ (mg g^‒1^)	1564.64
	K_S_ (L mg^‒1^)^ms^	0.003
	m_s_	0.40
	${\mathrm{R}}_{\mathrm{Adj}}^{2}$	0.97
	R^2^	0.98
	RSS	0.57
Hill	qH	185,675.49
	K_D_	36,684.36
	nH	0.40
	${\mathrm{R}}_{\mathrm{Adj}}^{2}$	0.97
	R^2^	0.98
	RSS	0.57
Redlich-Peterson	k_RP_ (L g^‒1^)	305,231.43
	a_RP_ (mg L^‒1^)^‒bRP^	60,301.59
	b_RP_	0.59
	${\mathrm{R}}_{\mathrm{Adj}}^{2}$	0.97
	R^2^	0.98
	RSS	0.56
Khan	q_s_ (mg g^‒1^)	0.14
	b_K_	6244.36
	a_K_	0.59
	${\mathrm{R}}_{\mathrm{Adj}}^{2}$	0.97
	R^2^	0.98
	RSS	0.57

K_F_ is a constant relating to the affinity of the adsorbate to the adsorbent. A large value of K_F_ in the Freundlich model implies the high affinity of Pb(II) for PG. Furthermore, 1/n value less than unity indicates the adsorption process is favorable.

Q_max_ in Langmuir isotherm indicates the monolayer adsorption capacity of PG and is a useful tool to compare different adsorbents for a specific contaminant. Table 4 shows the monolayer adsorption capacity of 9.47 mg/g in the present study was in the range or higher than many of those for low-cost adsorbents in the literature.

**Table 4:** Comparison of monolayer adsorption capacity for Pb(II) by different low-cost adsorbents.

Adsorbent	Q_max_(mg/g)	References	Adsorbent	Q_max_(mg/g)	References
Active carbon	6.68	^53^	Diatomaceous earth	8.5	^54^
Kaolin	4.50		Sporopollenin	8.5	^55^
Bentonite	7.56		Acacia nilotica	2.51	^56^
Blast furnace slag	5.52		Pinus pinaster bark	1.59–3.33	^57^
Fly ash	4.98		Tobacco stems	5.5–5.7	^58^
Perlite	8.906	^59^	Pottery granules	9.47	Present study

### Effect of interfering ions

**Figure 10:**
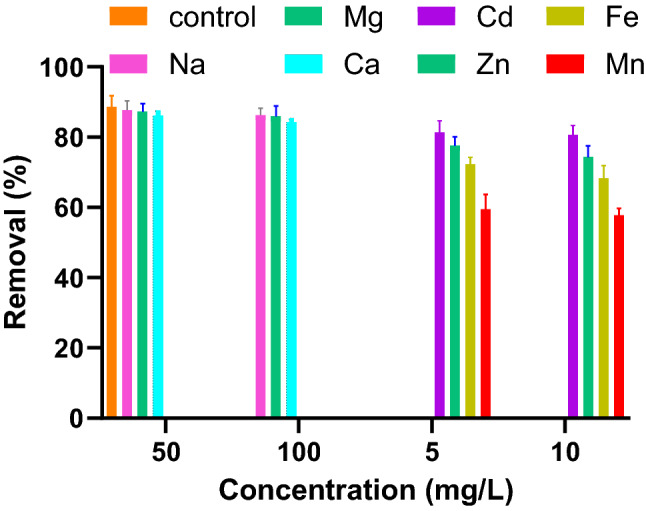
Effect of co-existing ions on Pb(II) adsorption by PG (adsorbent dosage: 10 g/L, Pb(II): 10 mg/L).

Natural waters always have a complex matrix of organic and inorganic impurities that affects the adsorbent-adsorbate interactions. The performance of the adsorption system needs to evaluate in the presence of major co-existing ions. Figure 10 illustrated how co-existing ions at the concentrations that are normally found in the water matrix affect Pb(II) adsorption. As seen, the level of inhibition by cationic ions decreased by manganese > iron > zinc > cadmium > magnesium > sodium. Furthermore, as the figure shows, increasing the ionic strength of any particular cation slightly affects the Pb(II) removal.

### PG adsorption cost

The cost of the adsorption system is a critical factor that determines the feasibility of the process, particularly in remote areas and low-income countries. One strategy to minimize the cost of the adsorption system is the use of waste materials or byproducts without or by minimal preparation steps. In particular, this approach led to the production of fewer waste streams and subsequent environmental and economic issues. Industries are increasingly under pressure to manage their wastes and valorization of the waste materials is also an interest from an industry viewpoint. The comparative cost analysis for low-cost and engineered adsorbents is not simply possible as it is rarely discussed in the literature. Previous reports classified adsorbents based on the origin, and the number of modification steps they required. A material could categorize as low-cost if the cost of preparation is lower than 1 $ per Kg^60^. PG used in this study was a cheap material obtained from the local pottery manufactories. The possibility of the use of a large amount of waste produced by pottery industries, and the absence of any chemical/thermal treatment, listed PG among the low-cost adsorbents.

## Conclusion

Present work reports the valorization of PG as a cheap, available, environmentally benign, and efficient sorbent for toxic Pb(II). Sorption experiments were conducted to determine how the physicochemical properties of the sorption system influence the Pb(II) adsorption. Fast adsorption and equilibrium time of about 30 min reflect the suitability of PG in real treatment systems. A higher adsorption efficiency by PG doses and mixing time observed is the result of more chance for Pb(II) to uptake by sorption sites. Alkaline pH and elevated solution temperature favored the adsorption process. Kinetic studies showed that the first-order kinetic model better describes the sorption process. Moreover, higher correlation factors for the Freundlich isotherm model, demonstrate multilayer adsorption of Pb(II) onto the heterogeneous surface of PG. The Freundlich constants also revealed the favorable nature of Pb(II) adsorption onto PG. Based on the nonlinear Langmuir model, the maximum monolayer adsorption capacity of PG was estimated to be 9.47 mg Pb(II)/g. The study of competitive co-current ions showed that the adsorption of Pb(II) was hindered significantly in the presence of Mn and followed in the order of Fe > Zn > Cd > Ca > Mg > Na.

## Data Availability

The datasets used and/or analyzed during the current study are available from the corresponding author on reasonable request.
